# Health care access of informal waste recyclers in Johannesburg, South Africa

**DOI:** 10.1371/journal.pone.0235173

**Published:** 2020-07-01

**Authors:** Jesne Kistan, Vusi Ntlebi, Felix Made, Tahira Kootbodien, Kerry Wilson, Nonhlanhla Tlotleng, Spo Kgalamono, Angela Mathee, Nisha Naicker

**Affiliations:** 1 Department of Community Health Medicine, Faculty of Health Sciences, University of Witwatersrand, Johannesburg, South Africa; 2 National Health Laboratory Services, National Institute for Occupational Health, Johannesburg, South Africa; 3 Department of Environmental Health, Faculty of Health Sciences, University of Johannesburg, Johannesburg, South Africa; 4 Environment and Health Research Unit, South African Medical Research Council, Johannesburg, South Africa; Jagiellonian University Medical College, POLAND

## Abstract

**Introduction:**

Informal waste recyclers contribute significantly to waste removal in South Africa. Waste recyclers face health hazards which are associated with handling and disposal of waste, a lack of personal protective wear and inaccessibility to occupational health care services. Consequently, accessing health care within the public health care sector is important for health outcomes in this population. This study assesses health care access of informal waste recyclers in South Africa to establish baseline information for health planning for potential inclusion of informal waste recyclers into occupational health services.

**Methods:**

A cross-sectional study of informal waste recyclers in two landfill sites in Johannesburg was conducted from March 2018. A standardized structured questionnaire was used to collect information on sociodemographic details, health care utilization, barriers to access and acceptability and affordability of health care. Factors associated with health care utilization were assessed using logistic regression.

**Results:**

A total of 363 informal waste recyclers were included in the study. Less than half of informal waste recyclers (41.0%) used health care facilities in the last 12 months. Those who accessed services chose to use facilities close to where they live (87.0%). Barriers to accessing health care services included long waiting periods (36.6%), being unable to take time off work (26.3%) and transport problems (13%). In the univariate analysis, factors such as gender and being treated well at the clinic and location of the health care facility were associated with health care utilization (OR: 1.97, p = 0.05, OR: 1.94, p = 0.02, OR: 0.65, p = 0.04 respectively).

**Conclusion:**

Informal waste recyclers face numerous challenges to accessing health care. Specific to their informal trade, barriers to health care utilization are related to financial repercussions due to the informal nature of their work.

## Introduction

Globally, informal waste recyclers are a vulnerable population who face occupational-related morbidities and mortality. Considered as the ‘invisible environmentalists’ of the world, waste recyclers contribute significantly to waste removal collection in many cities [1,2]. In South Africa, thousands of people generate livelihoods through the recycling of waste, in both the formal and informal sectors. In 2016, it was estimated that South Africa had approximately 51500 informal waste recyclers as compared to 5400 formal waste recyclers [3]. Despite the significant contribution of informal waste recyclers to the population, they remain neglected with regards to their health needs due to occupational hazards and lack of occupational health care.

Informal waste recyclers are those who do not form part of the formal employment of the municipal waste collection system. They earn a living from collecting, sorting and selling recyclable materials, such as paper, aluminum and plastics found mainly on the streets, in residential and commercial areas, or at landfill sites [4,5]. If informal waste recyclers in South Africa formed part of the municipal collection system, they would receive appropriate health care in the form of occupational health under the Occupational Health and Safety legislation of South Africa [6].

Waste recyclers face a range of hazards and health risks associated with handling and disposal of waste. Waste hazards may include chemical, biological and physical hazards [7]. In South Africa, a study conducted in nine landfill sites showed that health risks such as polluted water, rotten meat, faecal matter from soiled nappies, dust, exposure to heavy loads of waste, sharp objects such as needles and glass were prominent among informal waste recyclers [2].

Adverse health outcomes may be more prominent in waste recyclers than the general population due to their increased risk of exposure to hazardous substances such as heavy metal, faecal substances, medical waste and rodent infestation [8,9]. In a study conducted in India which compared non-waste recyclers to waste recyclers, waste recyclers were found to have significantly higher injuries (75%), respiratory illness (28%), eye infection (29%), and stomach problems (32%), compared to the comparison group (17%, 15%, 18%, and 19% respectively) [10]. Furthermore, a study in Brazil looking at waste recyclers showed a high prevalence of commonly reported diseases such as muscular disorders (78.7%); arboviruses (28.6%); episodic diarrhea (24.9%); hypertension (24.2%); bronchitis (14.3%); intestinal worms (12.6%) and diabetes (10.1%) [11]. The impact of poor health outcomes in informal waste recyclers and the inability to contribute to removal of environmental waste could result in increased spread of diseases in the general population.

The use of personal protective equipment (PPE) is essential in reducing the risk of injury and ill health associated with waste recycling. Informal waste recyclers may be particularly susceptible to waste recycling hazards since they do not have access to employer provided PPE [12].

Health care access within the public sector is critical for informal waste recyclers given their lack of access to occupational health services. It has been ascertained that health care access in South Africa is a challenge to many people (employed and/or unemployed) due to varying factors viz. social, financial, political etc. [13] Furthermore, waste recyclers may be particularly more vulnerable to health care access challenges due to the informal nature of their trade, their increased health risks and the lack of protection from occupational health care sectors. Access to health care is an important determinant of health outcomes of a population. Within the broader context of informal waste recyclers in South Africa, there is increasing pressure from worker movements to have informal waste recyclers become part of the formalized sector. A case needs to be made for informal workers to form part of the occupational health system to have access to protected health care. However, baseline information is first required as to how and why informal waste recyclers currently access health care in South Africa. This information could assist with health planning for possible inclusion of informal waste recyclers into occupational health services.

Little is known about health care access among informal workers in South Africa. Moreover, there is a scarcity of information regarding informal waste recyclers in South Africa unlike other developing countries such as India and Brazil where this group has received much attention [5,14,15]. While other studies have focused on the health risks that informal waste recyclers are exposed to, this study aims to evaluate health care access of informal waste recyclers in two of the four active landfill sites in Johannesburg, South Africa. This is in keeping with previous literature which has called for further research on the burden of disease of informal waste recyclers, spillover public health costs as well as the income and living standards impact on informal workers.

## Materials and methods

### Aim

To describe health care access of waste recyclers in Johannesburg, South Africa

### Study design and setting

A cross-sectional study was conducted on 363 participants who were conveniently recruited from two landfill sites in Johannesburg (Site 1: GPS Co-ordinates: -26.280993, 27.927661 and Site 2: GPS Co-ordinates: -26.191527, 27.880283) during the months of March and April 2018. Site 1 had a population of approximately 3000 waste recyclers and site 2, 600 recyclers [16].

### Study population and sample

Adult male and female informal waste recyclers who were actively working were included in the study. Convenient sampling was done since no worker lists were available and workers had unpredictable working times–characteristics which are common to the informal worker trade.

### Study procedures

Trained fieldworkers conducted interviews using a standardized structured questionnaire on a handheld device. The questionnaire was developed to assess the prevalence of health hazards, health outcomes and access to health care. The questionnaire (available as appendix I) was uploaded remotely onto RedCap—a data collection tool. Sociodemographic information was collected from all participants. Information such as age, gender, education level, household type, income and number of dependents was collected.

Health care service utilization was assessed by asking study participants about use of health care facilities within the past 12 months. Participants were also asked about the location of the facility that was used (i.e. close to where they lived, close to their work place or any facility). Affordability of health care services was evaluated by asking participants about the average amount of money that was spent on medical expenses per month (i.e. out of pocket payments for health care services). Acceptability of health care services was determined by asking participants whether they felt they were treated well at their health care facility, as well as whether they felt that they were discriminated against at their health care facility because of their trade as waste recyclers. Barriers to health care access by informal waste recyclers was assessed by asking participants about reasons for not accessing health care services. Participants could select more than one barrier to health care utilization. Sociodemographic factors, affordability and acceptability of health care services associated with health care utilization in the last 12 months was also evaluated.

### Statistical analysis

Data were imported from RedCap and cleaned prior to analysis. The data were analyzed using the statistical package, STATA 13.

Socio-demographic details were summarized. Continuous variables such as age, average monthly income and number of dependents were described using means and medians. Categorical factors such as education level and household type were described using proportions. Barriers were categorized into barrier present (if answered yes to any barrier) or barrier absent (if no barrier to health care utilization was selected). A multivariate logistic regression model was used to assess sociodemographic factors, barriers, affordability and acceptability of health care facilities associated with health care utilization type in the last 12 months. A univariate analysis was performed and factors with a significance level <0.05 were added to the final multivariate model. The model was also adjusted for confounding bias (site, gender) and interactions.

### Ethics

Written informed consent was taken from all participants. Ethics approval was given by the University of Witwatersrand Human Research and Ethics Committee, approval number: M171120. Permission was obtained from relevant authorities to access the landfill sites.

## Results

In total, 363 participants were included in the study (Table 1). The response rate was 99.5%. The mean age of all participants was 34 years old (range 18–81) and median was 31 years (IQR 27; 39). Majority of the cohort was male (73.0%), South African (81.2%), had received a secondary level education (77.9%) and resided in a back yard informal dwelling (30.3%). The average monthly household income was R1794.30 with a mean of three dependents per household. There were differences in the age, gender, education level, citizenship and number of dependents between the two sites. Cohorts from each site did not differ in monthly income and dwelling type. Acute illnesses or symptoms which participants sought treatment for included cough (41.1%), shortness of breath (27.1%), nausea or vomiting (23.5%), diarrhea (25.0%), headache (60.1%), fever (26.1%), muscle ache (21.8%) and itchy rash (47.4%) (Table 1).

**Table 1:** Socio-demographic details of informal waste recyclers per site and combined in Johannesburg, Gauteng.

	Sample (N)	Site 1 (N = 299)	Site 2 (N = 64)	P-value	Both Sites (N = 363)
**Age**	363			0.000	
Mean		32.1	42.9		34
Median (IQR)					31 (27; 39)
Range		18–65	18–81		18–81
**Gender**	363			0.000	
Male		235 (78.6%)	30 (46.9%)		265 (73.0%)
Female		64 (21.4%)	34 (53.1%)		98 (27.0%)
**Education level**	362			0.000	
No schooling		11 (3.7%)	4 (6.2%)		15 (4.1%)
Primary		35 (11.7%)	25 (39.1%)		60 (16.6%)
Secondary		248 (83.2%)	34 (53.1%)		282 (77.9%)
Tertiary		4 (1.4%)	1(1.6%)		5 (1.4%)
Missing	1				
**SA citizen**	361			0.000	
Yes		258 (86.0%)	35 (57.4%)		293 (81.2%)
No		42 (14.0%)	26 (42.6%)		68 (18.8%)
Missing	2				
**Dwelling type**	360			0.931	
Back yard dwelling—formal		57 (19.2%)	11(17.5%)		68 (18.9%)
Back yard dwelling—informal		91 (30.6%)	18 (28.6%)		109 (30.3%)
Formal house		85 (28.6%)	20 (31.7%)		105 (29.2%)
Informal dwelling		62 (20.9%)	13 (20.6%)		75 (21.0%)
Other		2 (0.7%)	1 (1.6%)		3 (0.8%)
Missing	3				
**Avg. Monthly Income (in ZAR)**	359			0.4162	
Mean		1754.8	1976.3		1794.3
Missing	4				
**Mean Number of Dependents**	360			0.000	
Mean		2.7	4.5		3
Missing	4				
**Acute Illnesses**					
Cough	168				69 (41.1%)
Shortness of Breath	70				19 (27.1%)
Nausea/Vomiting	85				20 (23.5%)
Diarrhoea	76				19 (25.0%)
Headache	148				90 (60.1%)
Fever	88				23 (26.1%)
Muscle Ache	142				31 (21.8%)
Itchy Rash	57				27 (47.4%)

**Fig 1:**
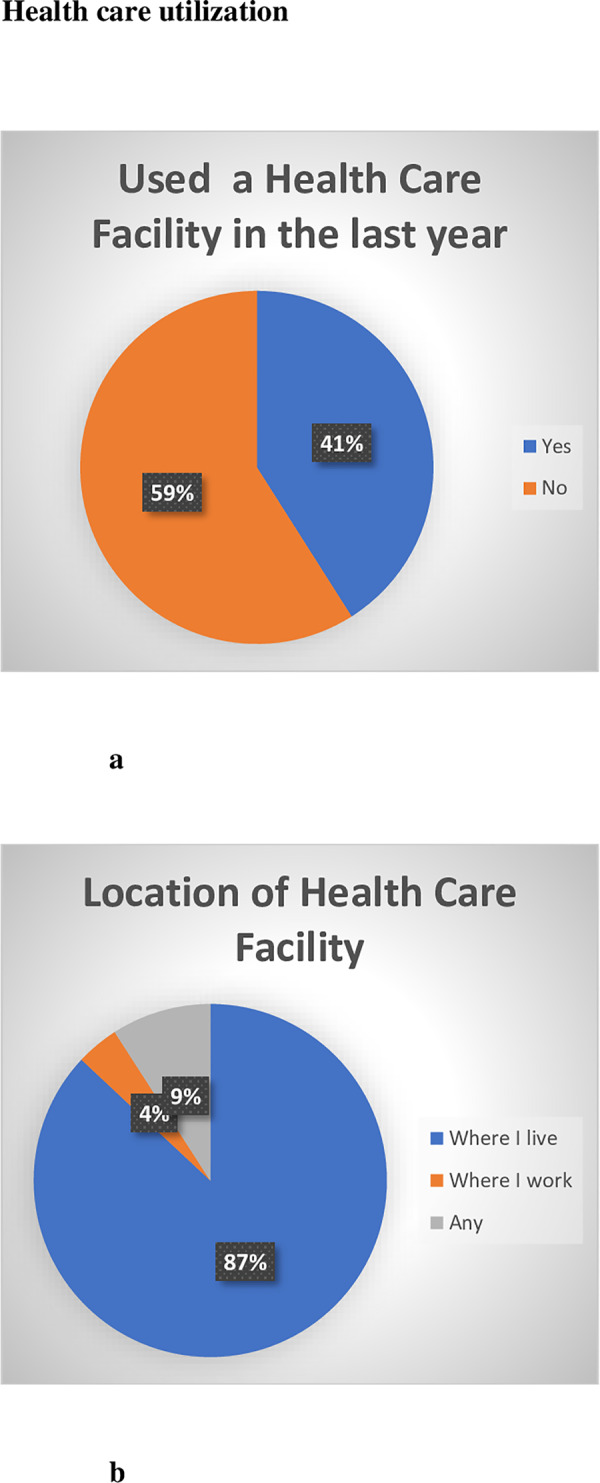
(a) Informal waste recyclers who accessed health care facilities in the last year (b) where informal waste recyclers access health care facilities.

Fig 1A and 1B show the health care service utilization of informal waste recyclers in Johannesburg. Less than half of informal waste recyclers (41.0%) used health care facilities in the last 12 months. However, the majority who accessed services chose to use facilities close to where they live (87.0%).

**Fig 2:**
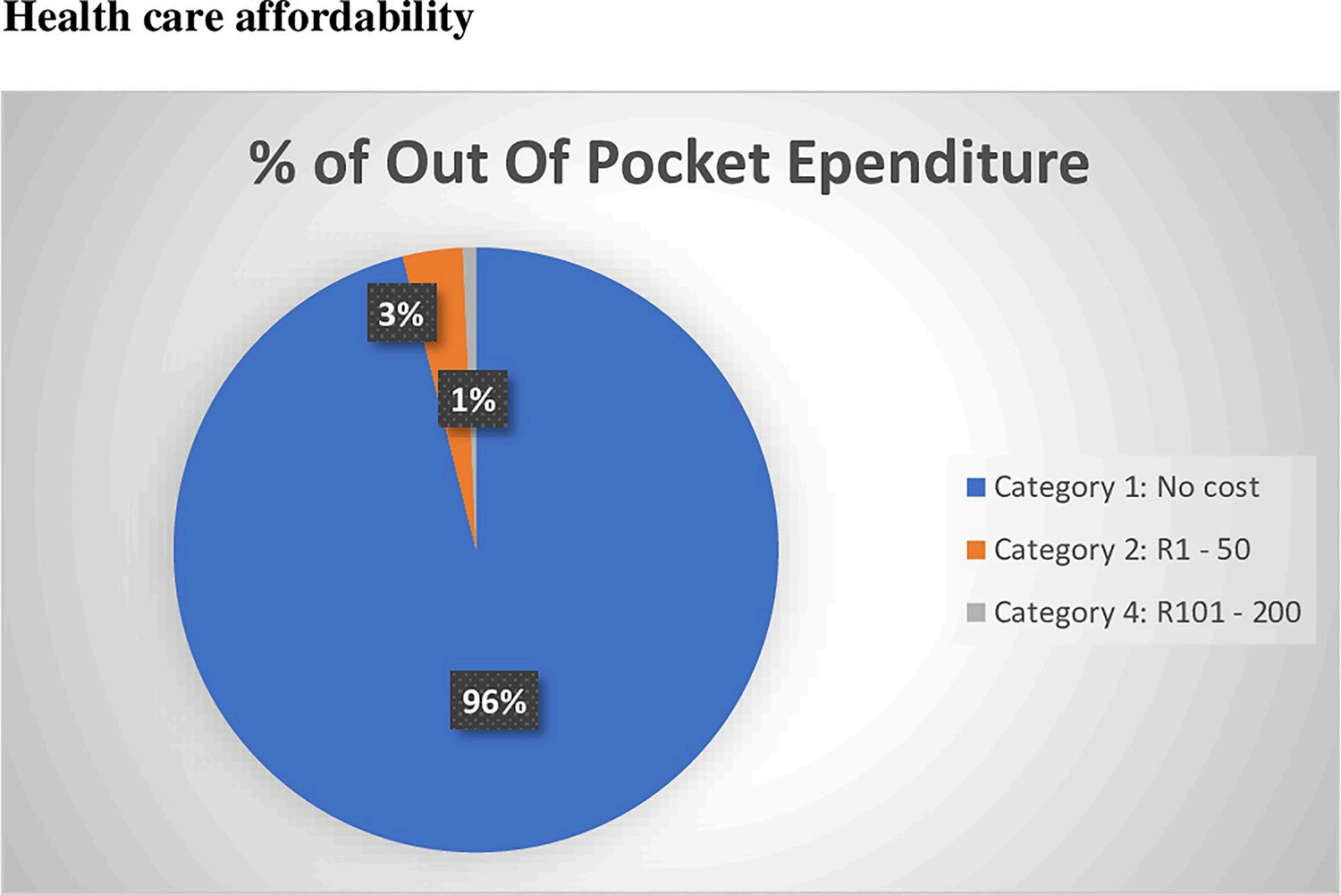
Out of pocket expenditure for health care services by informal waste recyclers in Johannesburg, Guateng.

Fig 2 shows out of pocket expenditure by informal waste recyclers in Johannesburg. For those that accessed health care service at any time, the majority of informal waste recyclers did not incur out of pocket expenditure for health care services (96.1%).

**Fig 3:**
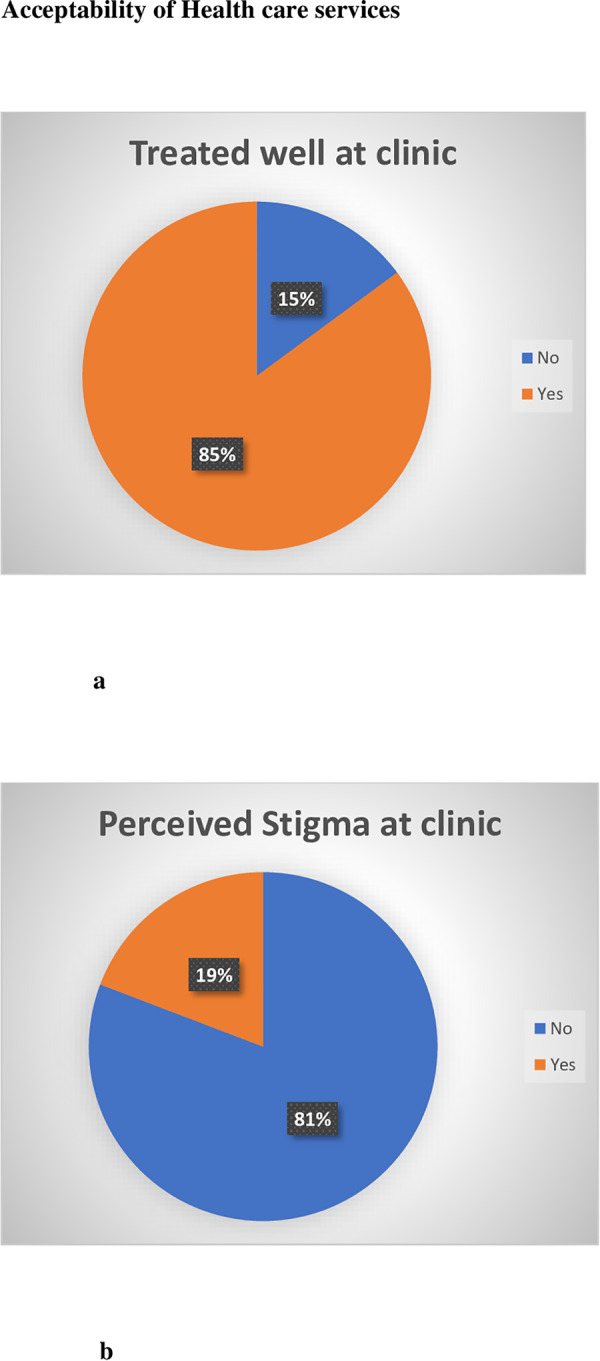
(a) Informal waste recyclers who felt they were treated well at the clinic (b) informal waste recyclers perceived stigma at the clinic.

Fig 3A and 3B shows acceptability of health care services reported by informal waste recyclers. Majority of informal waste recyclers felt that they were treated well by health workers when attending the clinic (85%). Almost 1 in 5 informal waste recyclers did feel that they were stigmatized by health care workers when attending the clinic.

Table 2 shows barriers to health care access reported by informal waste recyclers in Johannesburg. The greatest barriers to accessing health care services was long waiting period (36.6%), being unable to take time off work (26.3%) and transport problems (13%).

**Table 2:** Barriers to health care utilization of informal waste recyclers in Johannesburg, Gauteng.

Barrier	Number reported
Transport problems	47/361 (13.0%)
Unable to pay for services	31/361 (8.6%)
Unable to take time off work	95/363 (26.3%)
No health services where I live	12/361 (3.3%)
No health services where I work	13/361 (3.6%)
Problems getting child care	2/361 (0.6%)
Language problems	6/361 (1.7%)
Turned away from the clinic	4/361 (1.1%)
Poor quality of services or care	20/361 (5.5%)
Long waiting period	132/361 (36.6%)
Other	43/361 (11.9%)

A summary of the factors which were investigated for associations with health care utilization of informal waste recyclers in the last year is shown in Table 3. There was a significant association between gender and health care utilization. The odds of being male and utilizing health care was 1.97 times the odds of being female and utilizing health care in the last 12 months (p = 0.05). There was a significant association between being treated well at the clinic and health care facility utilization (p = 0.02). In the multinomial logistic regression model, there was a significant association of being male and health care utilization (OR: 1.74, p = 0.04). There were no other significant associations with utilizing health care facilities.

**Table 3:** Factors associated with accessing healthcare in the last 12 months (Univariate and multivariate models).

	Univariate		Multivariate	
Factor	OR (95% CI)	P-value	Or (95%)	P-value
Age	1.01 (0.99; 1.04)	0.162		
Gender (Male Vs. Female)	1.97 (1.23; 3.16)	0.005*	**1.73 (1.03; 2.91)**	**0.040**
Education level	0.76 (0.52; 1.11)	0.164		
SA citizen (Yes Vs. No)	1.03 (0.60; 1.78)	0.895		
Out of Pocket Expenditure	1.50 (0.68; 3.30)	0.311		
Location of Healthcare Facility	0.65 (0.44; 0.97)	0.036*	0.72 (0.48; 1.10)	0.130
Being treated well at the clinic (No Vs. Yes)	2.62 (1.16; 4.38)	0.015*	1.94 (0.98; 3.85)	0.058
Perceived Stigma at Clinic (No Vs. Yes)	1.15 (0.66; 2.00)	0.615		
Barrier (Yes Vs. No)	1.41 (0.86; 2.30)	0.171		
Site (Site 1 Vs. Site 2)	1.04 (0.60; 1.83)	0.871	0.78 (0.41; 1.42)	0.398

## Discussion

Under half of the informal waste recyclers utilized health care facilities in the last 12 months. Many of the waste recyclers suffered varying acute illnesses (21.8% - 60.1%) such as cough, shortness of breath and headache but under half (41.0%) chose to use a health care facility.

Our study further explored barriers as to why people did not access health care. The most common barrier to utilizing health care was long waiting periods. This is not only common to informal waste recyclers and may be generalizable to the population seeking public health care [13,17]. In other countries, informal workers have also expressed long waiting periods and fruitless trips to health care facilities as a deterrent to seeking health care [15]. This may be due to the direct effect of a day missed at work equals a loss of income. The next most common barrier noted was being unable to take time off. Unlike those in the formal sector, informal waste recyclers are not protected by occupational legislation which entitles them to income-protected time off for health care. Transport problems were also identified as barriers to utilizing health care. Transport is costly and health facilities may be located further from where waste recyclers live and work. Occupational health facilities for formal workers are often located at the workplace and this may negate the difficulty of requiring transport to utilize health care. If informal workers were allowed access to onsite occupational health services, health care service utilization may improve, barriers to accessing health care could be avoided and improved health care outcomes as a result of early disease diagnosis and treatment may be achieved.

Informal waste recyclers who did access facilities, used facilities which were closest to where they lived. This may emphasize the need to have clinics close to areas where people reside. This is in keeping with previous studies done in South Africa which highlighted the importance of availability of health services close to where people reside and work [13]. By introducing occupational health clinics to informal waste recyclers, informal waste recyclers may choose to access on site health facilities. This could enable better health seeking behavior, targeted and appropriate medical screening and treatment as well as lessen the burden on the public health care system.

Gender was also associated with health care utilization. In a large study conducted in Gauteng, South Africa in 2013, higher odds (OR 2.18, 95% CI: 1.88–2.53, p = 0.001) of health care utilization was associated with being female [18]. This differed from our study which showed higher odds of health care utilization being associated with male gender. Our study may differ from the general population, as males in this sector may perceive themselves to be at a higher risk of poor health outcomes due to the occupational hazards faced and thus, utilized services more readily. However, gender as a determinant of health care utilization may be a moot point if occupational health care services are extended to informal waste recyclers.

The majority of informal waste recyclers felt that they were treated well at the local clinic (Fig 3A). This differed from other studies in South Africa which showed that patients felt that they were poorly treated at their health care facility [13,19]. Harris et al showed that over half of all respondents (54.7 per cent) felt that patients at public hospitals are ‘rarely treated with respect and dignity’ [13]. However, almost one in five waste recyclers felt that they were discriminated against by the clinic. Informal workers may further be susceptible to discrimination due to their trade. In qualitative studies conducted with informal workers, negative attitudes of health workers towards clients were reported [20]. Other studies have showed that health care inequalities exist between formal and informal workers even when controlling for sex and age. Informal workers tend to have worse self-reported health statuses and experience more difficulty in accessing health care [21]. Discrimination of informal waste recyclers must be avoided by occupational health care workers to ensure health care utilization of occupational clinics if these services are extended to the informal sector.

Most informal waste recyclers did not incur out-of-pocket expenses for medical treatment. This may be due to the fact that most recyclers used free primary health care facilities that were close to where they lived (transport costs were not incurred). Moreover, most informal waste recyclers, chose to use primary care clinics as the first port for seeking health care (data not shown). Two of the waste recyclers did incur costs of between R100-200 per month for medical expenditure. With a median household income of R1500 per waste recycler, this could amount to almost 7–13% of spending on health care which could constitute a serious financial burden for waste recyclers and their households. Our study highlights that informal waste recyclers may not be prepared to spend money on health care services (such as general practitioners or private insurance) despite having greater health risks than the general population. Occupational health services which are provided for free may further enhance the health seeking behavior of informal waste recyclers.

The limitations of the study included recall bias and interview bias which informal waste recyclers may have faced when answering questions about past health care experiences. The cross-sectional study design and convenient sampling may have also limited the study. This was a quantitative study with a structured interview so further questions on barriers to health care were not able to be ascertained at the time of data collection.

The impact of under-utilization of health care services and work-related barriers to accessing health care services could result in poorer health outcomes due to delayed help seeking behavior, delayed diagnosis and treatment of disease. In South Africa, waste removal is heavily dependent of both the formal and informal sector. Attrition of the informal sector could lead to unhealthy environments, increased spread of diseases in the population and damage to the general environment. The impact of poor health in this population has a cascading effect on the general population at large. Therefore, measures must be taken to address health care utilization and health care access in vulnerable occupational groups.

Occupational health services which are provided onsite, free and in a non-discriminatory manner will improve health utilization for informal waste recyclers.

## Conclusion

Informal waste recyclers face similar challenges to accessing health care as the general population. They access health services close to their places of residence, rather than work. However specific to their informal trade, barriers to health care utilization are related to financial repercussions due to the informal nature of their work i.e. long waiting periods and being unable to take time off work resulting in loss of income. If informal waste recyclers are not able to readily access health care services, it may result in poor health outcomes in this group, resulting in decreased waste recycling at a population level that impacts both the environment and communities. Introducing occupational health services to informal workers needs to be explored. Several of the barriers found to hinder health care services may be obliterated by the formalization of this sector and the inclusion into occupational health services.
